# Genotyping of *Toxoplasma gondii* in domestic animals from Campeche, México, reveals virulent genotypes and a recombinant *ROP5* allele

**DOI:** 10.1017/S0031182024000106

**Published:** 2024-04

**Authors:** Claudia Patricia Rico-Torres, Luis Fernando Valenzuela-Moreno, Elvira Robles-González, Alvar Alonzo Cruz-Tamayo, Miguel Huchin-Cab, Jonathan Pérez-Flores, Lizbeth Xicoténcatl-García, Héctor Luna-Pastén, Luz Belinda Ortiz-Alegría, Irma Cañedo-Solares, Carlos Cedillo-Peláez, Fernando García-Lacy, Heriberto Caballero-Ortega

**Affiliations:** 1 Laboratorio de Inmunología Experimental, Instituto Nacional de Pediatría, México; 2 Facultad de Ciencias Agropecuarias, Universidad Autónoma de Campeche, México; 3 Departamento de Observación y Estudio de la Tierra, la Atmósfera y el Océano, El Colegio de la Frontera Sur, México; 4 Facultad de Medicina Veterinaria y Zootecnia, Universidad Nacional Autónoma de México, México

**Keywords:** Campeche, genotyping, México, PCR-RFLP, *Toxoplasma gondii*, virulence

## Abstract

*Toxoplasma gondii* has at least 318 genotypes distributed worldwide, and tropical regions usually have greater genetic diversity. Campeche is a state located in the southeastern region of México and has favourable climate conditions for the replication and dissemination of this protozoan, similar to those in South American countries where broad genetic diversity has been described. Thus, in this study, 4 *T. gondii* isolates were obtained from tissues of stray dogs and free-range chickens in Campeche, México, and were genotyped by Mn-PCR-RFLP with 10 typing markers (*SAG1*, *altSAG2*, *SAG3*, *BTUB*, *GRA6*, *c22-8*, *c29-2*, *L358*, *PK1* and *Apico*) and 5 virulence markers (*CS3*, *ROP16*, *ROP17*, *ROP18* and *ROP5*) to provide new information about the distribution and virulence prediction of *T. gondii* genotypes. Two isolates of *T. gondii* genotype #116 and 2 of genotype #38 were obtained from stray dogs and chickens, respectively. The parasite load found in these species was between <50 and more than 35 000 tachyzoites per mg of tissue. Virulence marker genotyping revealed a recombinant 1&3 *ROP5* RFLP pattern in 2 ToxoDB #116 isolates with no prediction of virulence in a murine model, while in the 2 ToxoDB #38 isolates, the *ROP18*/*ROP5* combination predicted high virulence. Considering all the typed markers, there is a predominance of type I and III alleles, as constantly reported for the isolates characterized in various regions of México. It is crucial to determine their phenotype to corroborate the genetic virulence profile of the *T. gondii* isolates obtained in this study.

## Graphical abstract

**Figure:**
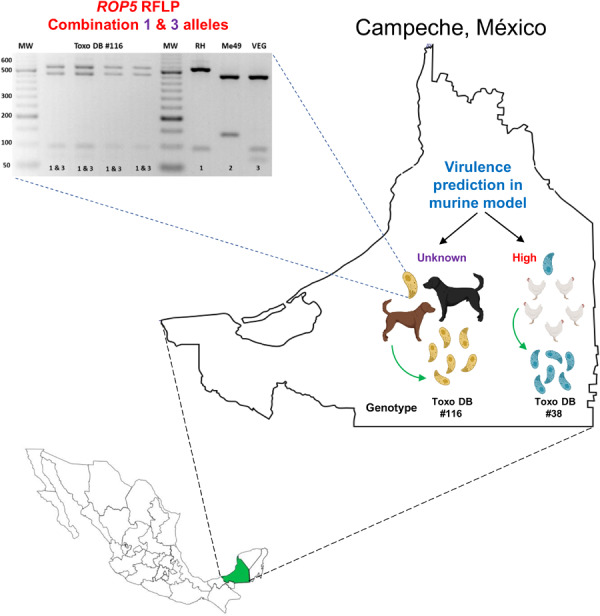

## Introduction

*Toxoplasma gondii* is an Apicomplexa parasite distributed worldwide that is responsible for toxoplasmosis, a disease of great medical and veterinary relevance. The parasite is capable of infecting all homeothermic organisms, including humans (Delgado *et al*., 2022). The prevalence of *T. gondii* in México is 43.9% in the human population, with the Neotropical region having the highest rate of antibodies against this parasite (Caballero-Ortega *et al*., 2012). The interest in characterizing this protozoan in the Neotropical region of México is due to the combination of climatic and biological factors that favour the wide genetic diversity of *T. gondii*, as has been reported in several South American countries.

To date, at least 318 genotypes have been reported worldwide, and tropical regions usually have greater genetic diversity (Shwab *et al*., 2014; Meireles *et al*., 2022). Previous reports about the genotyping of *T. gondii* isolates obtained from feral cats and stray dogs from Quintana Roo and Chiapas, respectively, revealed the great genetic diversity of this protozoan and strong infection pressure, with individuals infected with up to 5 different *T. gondii* strains in the same urban environment shared by different species, including humans (Valenzuela-Moreno *et al*., 2019, 2020, 2022*b*). Campeche is a southeastern Mexican state that has favourable climatic conditions for the replication and dissemination of this protozoan, since 80% of its territory is rainforest. More than 4379 species of vertebrate animals live in this area, of which the family Felidae is represented by the domestic cat as well as by 5 of the 6 species of wild felines reported for México (Guzmán-Soriano *et al*., 2013; CONABIO, 2021). However, studies of this zoonotic agent in this state are scarce. To date, there is only one study that attempted to isolate *T. gondii* DNA in tissues of wild bat (*n* = 25) from the State of Campeche, where all of the analysed animals were negative for the *B1* gene by PCR (Torres-Castro *et al*., 2019). Therefore, the aim of this work was to identify, isolate and genotype *T. gondii* in sentinel hosts such as dogs (*Canis familiaris*) and free-range chickens (*Gallus gallus*) from Campeche, México, to determine which genotypes are circulating in this particular region and to predict the degree of virulence using an extended panel of 15 biomarkers assayed by the polymerase chain reaction-restriction fragment length polymorphism (PCR-RFLP) approach.

## Materials and methods

### Tissue samples

From February to June 2021, stray dogs and free-range chickens from Escárcega (18°37′1″ N, 90°43′1″ W) and Calakmul (19°12′–17°48′ N, 89°09′–90°28′ W), respectively, both municipalities of Campeche, were captured and euthanized to collect blood and tissue samples. These counties were located 107 km apart from each other. The body condition of dogs was evaluated according to the Purina® Body Condition Tool (http://www.purina.co.uk), which ranges from 1 (severely underweight) to 9 (clinically obese), where 5 is considered the ideal score. Samples from both animal species were collected in collaboration with the Facultad de Ciencias Agropecuarias of the Universidad Autónoma de Campeche (UAC), México, and the Departamento de Observación y Estudio de la Tierra, la Atmósfera y el Océano, of Colegio de la Frontera Sur (ECOSUR). The stray dogs were anaesthetized with tiletamine-zolazepam (7.5 mg kg^−1^ body weight, Zoletil-Virbac®, México), and once a dog was placed on an anaesthetic plane, 12 mL of whole blood was obtained from the cephalic vein for DNA extraction. Subsequently, an intravenous overdose of sodium pentobarbital (25 mg kg^−1^, Dolethal®, Vetoquinol, France) was administered to cause cardiorespiratory arrest and complete the euthanasia procedure. Free-range chickens were euthanized by cervical dislocation. Euthanasia procedures were performed in accordance with the guidelines of the Mexican Official Standard NOM-033-SAG/ZOO-2014 and Law for the Protection and Welfare of Animals of the State of Campeche. Necropsies were carried out following the methodology described by Schuneman and Constantino (2002), and samples of heart, lung, liver, spleen, striated muscle (diaphragm and right gracilis) and brain from both species were obtained to attempt molecular diagnosis, isolation of the parasite by bioassay in mice, and histopathological analysis.

### ELISA

Detection of IgG anti-*T. gondii* in dogs was carried out as previously described with slight modifications (Cedillo-Peláez *et al*., 2012). Briefly, ELISA plates (NUNC Maxisorb) were sensitized with 100 μL of crude extract of tachyzoites from *T. gondii* strain RH (type I) (2 *μ*g mL^−1^). Sera and goat anti-dog IgG H&L (HRP) (Abcam, NL, ab112852) were diluted 1:200 and 1:8000 in 0.05% PBS-T20, respectively. Detection of the antigen–antibody immunocomplexes was carried out with a chromogen–substrate solution for peroxidase (H_2_O_2_/O-phenylendiamine Sigma-Aldrich, MA, USA). The reaction was stopped with 50 μL of 1 N sulphuric acid. Negative and positive sera from previously tested dogs were included in each plate (Cedillo-Peláez *et al*., 2012). The cutoff point was calculated by obtaining the average absorbance of the negative controls plus 3 standard deviations, and the reactivity index (RI) was calculated by dividing the absorbance of each serum sample by the cutoff point. Sera with IR >1.0 were considered positive. Detection of anti-*T gondii* antibodies in chicken sera was not performed since no blood samples were obtained.

### Western blot

Immunoblotting was carried out as previously described and adapted to test dog sera (Caballero-Ortega *et al*., 2008). In brief, *T. gondii* proteins from 20 million tachyzoites of the RH strain (type I) were separated by electrophoresis. The proteins were electrotransferred to a nitrocellulose membrane, strips of 3 mm width were cut, and serum samples diluted 1:200 in PBS-T20 were added. Then, goat anti-Dog IgG H&L (HRP) diluted 1:2000 in PBS-T20 was added. Immune complexes were detected using a substrate/chromogen solution with 4-chloro-1-naphthol (Sigma-Aldrich, MA, USA). Sera were considered positive when at least 3 diagnostic bands were detected on the nitrocellulose membrane.

### Bioassay in mice

Parasite isolation was carried out under the NOM-062-ZOO-1999 guidelines for the handling and care of mice and as previously described (Rico-Torres *et al*., 2015). Briefly, the brain and pooled heart and diaphragm tissue from each dog or free-range chicken were manually macerated in a mortar in sterile PBS (pH 7.2) with 10 000 IU mL^−1^ penicillin G. Then, 1.0 mL homogenate was used to intraperitoneally inoculate two BALB/c mice per dog or chicken. All inoculated mice were examined daily for 45 days for signs of *T. gondii* infection. Animals with severe signs of illness, such as bristly hair, lethargy, ascites, loss of body weight and neurological signs, were euthanized, and imprints of the lung and brain were observed under a compound microscope to search for tachyzoites or tissue cysts (Fernández-Escobar *et al*., 2021). One week after inoculation, peritoneal lavages with sterile PBS were performed to obtain tachyzoites and proliferate them in NIH 3T3 mouse fibroblast culture (ATCC® CRL-1658™), as previously described (Khan and Grigg, 2017). The surviving mice were euthanized 45 days after inoculation and were considered infected if tachyzoites or tissue cysts were detected in any of their tissues.

### DNA extraction

Peritoneal exudates with apicomplexan parasites from infected mice, blood buffy coat and tissue samples from dogs and chickens were subjected to DNA extraction (Brenier-Pinchart *et al*., 2015) following the manufacturer's instructions (Qiagen Gentra® Puregene® Tissue Kit, Hilden, Germany). The DNA obtained was quantified with a spectrophotometer (Thermo Scientific Nanodrop™ 1000, MA, USA) and kept at −20°C until use.

### PCR and genotyping

To detect *T. gondii* DNA in the tissue samples and peritoneal exudates, endpoint and real-time PCR were performed to amplify the 200 to 300-fold repetitive 529 bp non-coding sequence -SeqRep529- (Tox4-5) and 35-fold repetitive *B1* gene. Parasite burden in tissues was also determined by standard curves generated by spiking 50 to 5 × 10^6^ tachyzoites of the RH strain in tissues of uninfected mice. Data were analysed using the Step One software 2.0. The linear regression coefficients (*R*^2^) and amplification efficiencies were calculated (Homan *et al*., 2000; Kompalic-Cristo *et al*., 2007; Cedillo-Peláez *et al*., 2011). The isolates of *T. gondii* obtained were also tested by endpoint PCR for the SeqRep529 marker. Positive samples for at least one of the molecular assays were included in a multilocus nested PCR (Mn-PCR-RFLP) to amplify 10 polymorphic markers: *SAG1*, alt. *SAG2, SAG3, BTUB, GRA6, c22-8, c29-2, L358, PK1* and *Apico* (Su *et al*., 2010). Additionally, clinical samples and DNA from *T. gondii* isolates were included in the Mn-PCR-RFLP to genotype *CS3*, *ROP16*, *ROP17*, *ROP5*, and *ROP18* virulence markers as previously described (Pena *et al*., 2008; Shwab *et al*., 2016). All PCR assays (real time and endpoint) were carried out with AmpliTaq Gold™ polymerase (Thermo Fisher Scientific, cat. 4311806, MA, USA). Internal and external primers of the *CS3* marker had the same melting temperature (*t*_m_) as the oligonucleotides of the *ROP* markers; therefore, they were included together in the Mn-PCR-RFLP. All amplicons were digested with specific restriction enzymes (New England, Biolabs®, USA and Thermo Scientific®, USA) as previously described (Pena *et al*., 2008; Su *et al*., 2010). DNA samples of strains representative of the three archetypal lineages, RH, Me49 and VEG (*Toxoplasma gondii* ATCC® 50611 ™ and 50861™) reference strains (types I, II and III, respectively), were included as positive controls, while sterile water was used as a negative control. Nested PCR products were resolved in 1.5% agarose-TBE gels stained with ethidium bromide (EtBr). Additional controls were added to ensure that there was no DNA contamination (positive and negative reamplification controls). All restriction products were resolved in 3% agarose-TBE gels stained with EtBr and digitalized.

### Histopathology and immunohistochemistry (IHC)

Tissues fixed in 10% buffered formalin were processed for analysis by the conventional technique for histology and stained with hematoxylin and eosin. For immunohistochemistry, samples were processed using the streptavidin–biotin–peroxidase complex (Histostain-Plus, Invitrogen, USA) as previously described, with slight modifications (Valenzuela-Moreno *et al*., 2022*a*). Briefly, paraffin-embedded sections were cut and mounted on electrocharged slides (Kling-On-Hier, Biocare). Subsequently, the tissues were incubated with serum from *T. gondii*-positive mice experimentally infected with the *T. gondii* Me49 strain (dilution 1:300). The slides were then incubated with a secondary multispecies biotinylated antibody (Invitrogen, USA), followed by incubation with streptavidin-peroxidase. Immune complexes were revealed with the commercial Betazoid DAB solution, chromogen 3,3′ diaminobenzidine (Biocare®). Liver and spleen sections of mice infected with *T. gondii* strain Me49 were used as positive controls, and the primary antibody was replaced with PBS as a negative control. Histological and IHC sections were examined by light microscopy (Zeiss Axiostar plus; Göttingen, Germany).

## Results

Eleven stray dogs were captured, all of which were adults (>1 year old). Their body condition ranged from severe to slightly underweight; externally, most of the stray dogs had skin diseases (scabies) and other ectoparasite infestations (fleas and ticks not identified) of varying degrees. No lesions compatible with acute or chronic *T. gondii* infection were found during necropsy and histopathological analyses. In the brain of dog #8, only one *T. gondii*-immunopositive tissue cyst was observed. The frequency of IgG anti-*T. gondii* antibodies found in dogs by ELISA and Western blot was 72.7% and 100%, respectively.

### Molecular identification and parasite load

Six out of eleven dogs (54.5%) were positive in at least one tissue sample according to endpoint PCR and qPCR. The parasite load was between 50 and 4025 tachyzoites/mg of tissue. Two *T. gondii* isolates from dog #8 were obtained, one from the heart/diaphragm pool (TgDogMxCam8a) and one from the brain (TgDogMxCam8b), both showing the ToxoDB #116 genotype. In addition, in the spleen of the same dog, mixed infection (I + II + III) was detected for the *SAG3* gene (Table 1).

**Table 1.** PCR-RFLP genotypes from clinical samples and *Toxoplasma gondii* isolates obtained from stray dogs and free-range chickens in Campeche State, Mexico

ID	Host	Municipality	Tissue/Isolate	*B1 gene* qPCR	Tachyzoites/mg tissue	Genetic markers+										ToxoDB PCR-RFLP genotype
						*SAG1*	*altSAG2*	*SAG3*	*BTUB*	*GRA6*	*c22-8*	*c29-2*	*L358*	*PK1*	*Apico*	
TgDogMxCam1	Dog 1	Escárcega	Diaphragm	+	96		I	+		I			III	I		
TgDogMxCam4	Dog 4		Brain	+	<50			+								
TgDogMxCam5	Dog 5		Brain	+	<50			+			III					
			Spleen	+	<50			III								
TgDogMxCam8	Dog 8		Diaphragm	+	4025	I	III	III	I	III	II		III	III	+	Likely #116
			Spleen	+	<50			I + II + III								
			Isolate a*	ND	ND	I	III	III	I	III	II	III	III	III	III	#116
			Isolate b*	ND	ND	I	III	III	I	III	II	III	III	III	III	#116
TgDogMxCam10	Dog 10		Heart	+	287	I		+		I						
			Spleen	+	73			+		I						
TgDogMxCam11	Dog 11		Spleen	+	<50			+								
TgCkMxCam1	Chicken 1	Calakmul	Heart	+	35,793	I	III	III	I	I	I	III	I	I	III	#38
			Isolate a*	ND	ND	I	III	III	I	I	I	III	I	I	III	#38
			Isolate b*	ND	ND	I	III	III	I	I	I	III	I	I	III	#38

*Isolates obtained from the same animal were designated as ‘a’ and ‘b’ (heart and brain, respectively). +The expected amplicon was obtained but could not be subjected to RFLP. ND: Not done.

Seven free-range chickens were sampled, all of them were adults. Two *T. gondii* isolates (TgCkMxCam1a and TgCkMxCam1b) from the heart and brain of chicken #1 were obtained, and both were the ToxoDB #38 genotype. The parasite load found in this chicken was more than 35 000 tachyzoites per mg of tissue (Table 1).

### Genotyping of virulence markers

**Figure 1.**
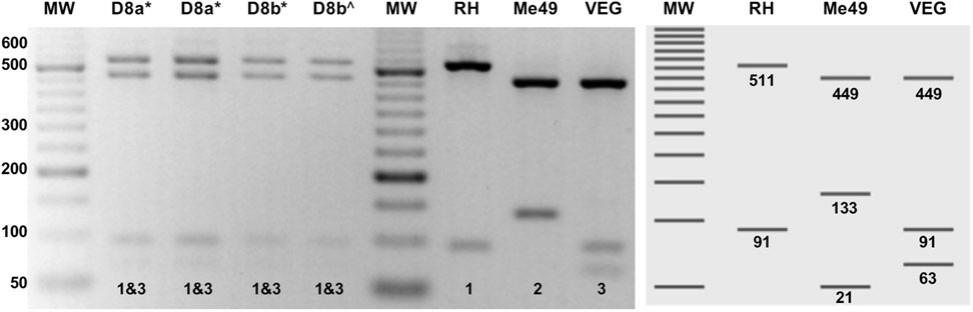
Representative PCR-restriction fragment length polymorphism pattern of *T. gondii ROP5* determined in TgDogMxCam8. A novel *ROP5* locus showing a recombinant 1&3 *ROP5* RFLP pattern in one dog from Escárcega, Campeche. D8a: TgDogMxCam8a; D8b: TgDogMxCam8b. PCR-RFLP was performed on peritoneal lavage (*) and cell culture (^) samples. Virtual digestion was performed with the Benchling digestion tool (www.benchling.com). RH, Me49 and VEG are type I, II and III reference strains, respectively. MW: molecular weight marker of 50 bp.

The two isolates from dog #8 have alleles III and 3 for *CS3* and *ROP18*, respectively, while *ROP16* and *ROP17* have type 1 alleles (Table 2). For the *ROP5* locus, a different restriction pattern was found (Fig. 1); it resembled that of the combination of types 1 and 3. Although the combination of alleles at the *CS3* and *ROP18* loci predicts low virulence in mice for the two TgDogMxCam8 isolates, the true virulence prediction *ROP18/ROP5* is unknown due to the combination of type 1 and 3 alleles of *ROP5*; alleles 1 and 3 contain a virulent cluster, while allele 2 is avirulent. The partial genetic characterization of the virulence markers of a clinical sample from dog #1 showed that this could be a highly virulent strain of *T. gondii* (*ROP18*/*ROP5* 4/1) in the mouse model (Table 2).

**Table 2.** Virulence multilocus PCR-RFLP typing of clinical samples and *Toxoplasma gondii* isolates from stray dogs and free-range chickens in Campeche State, México

ID	ToxoDB genotype	Virulence genetic markers					Virulence based on bioassay dead/inoculated	Virulence prediction in mouse bioassay+
		*CS3*	*ROP16^&^*	*ROP17^*	*ROP18*	*ROP5*		
RH	#10	I	1	1	1	1		High virulence
Me49	#1	II	2	2	2	2		Low virulence
VEG	#2	III	1	2	3	3		Low virulence
Present study								
Dog #1 (diaphragm)	-	NA	1	NA	4	1	&	High virulence
Dog #8 (diaphragm)	#116	III	1	1	3	1&3	&	♦Low virulence?
TgDogMxCam8a*	#116	III	1	1	3	1&3	1/1	
TgDogMxCam8b*	#116	III	1	1	3	1&3	1/1	
Chicken #1 (heart)	#38	I	1	1	1	3	&	High virulence
TgCkMxCam1a*	#38	I	1	1	1	3	1/1	
TgCkMxCam1b*	#38	I	1	1	1	3	1/1	

*Isolates obtained from the same animal were designated as ‘a’ and ‘b’ (heart and brain, respectively). ^&^Strains of type I and III have allele 1. ^Strains of type II and III have allele 2. +Based on Shwab *et al*. (2016). ^&^Genotyping was performed from original tissues of the host. ♦Given the possible *ROP18*/*ROP5* combinations (3/3 and 3/1), it could be predicted as a low-virulence strain.

Considering the combination of results obtained for the virulence markers, particularly for *ROP*18/*ROP*5 1/3, high virulence was predicted in the murine model for the 2 TgCkMxCam1 isolates (Table 2). All blood samples from the included individuals were negative for *T. gondii* DNA detection.

## Discussion

In México, there are multiple reports regarding *T. gondii* frequency in wild and domestic species, including dogs, animals that are usually in close contact with humans and can serve as sentinels of its infection pressure. Chickens have also been used to demonstrate the presence of the parasite in a given region due to their ground feeding habit (Costa *et al*., 2021). However, to date, there have been few studies reporting the infection of this parasite in Campeche. Therefore, the objective was to identify and genetically characterize the parasite in tissues of stray dogs and free-range chickens living in Campeche.

The high frequency of IgG anti-*T. gondii* antibodies found in dogs (72.7% and 100% by ELISA and Western blot, respectively) and molecular detection results for *T. gondii* DNA reflect the high infective pressure in which the individuals from Campeche, as was reported a few years ago in the human population of this state (Caballero-Ortega *et al*., 2012). These results show the high infection pressure of *T. gondii* in urban and rural environments shared by different species, including humans. In this study, none of the tested animals were positive for the presence of *T. gondii* DNA in peripheral blood, in contrast to the results obtained in other southeastern states (Valenzuela-Moreno *et al*., 2019, 2020). This was probably due to the season of the year (November and December) in which the samples were collected in Quintana Roo and Chiapas, months when the average rainfall is higher, and the oocysts can be transported into and stagnate in puddles where stray animals can drink them (CONAGUA, 2023).

DNA of *T. gondii* was determined in some tissues of the captured animals, and genotyping was achieved (partially in some cases), including those from which the 4 isolates were obtained. Although few alleles could be typed in most of the clinical samples, triple infection (*SAG3* alleles I + II + III) was detected in the spleen of dog #8, a result that is quite common in the southeastern region of México and has also been reported in cases of congenital toxoplasmosis in the central zone of the country (Rico-Torres *et al*., 2018; Valenzuela-Moreno *et al*., 2019, 2020). In tissues where *T. gondii* isolates were obtained, almost all genetic markers were genotyped, and the genetic profiles of the clinical sample and the isolate were correlated, suggesting that these tissues had a higher parasite load.

Six isolates of *T. gondii* had previously been obtained from chickens in the central region of México (Dubey *et al*., 2004). Four out of 6 of these isolates were the archetypal type III clone (ToxoDB #2). Several isolates have also been obtained from dogs, demonstrating 4 different genotypes (ToxoDB #8, #28, #73 and #74) (Dubey *et al*., 2009; Valenzuela-Moreno *et al*., 2020; Rico-Torres *et al*., 2023). Similarly, we described the ToxoDB #116 genotype in 2 Bennett's wallabies (*Macropus rufogriseus*) from the centre plateau of México that had died due to acute disseminated toxoplasmosis. This genotype has also been reported in Argentina, Brazil, Peru and Venezuela (Dubey *et al*., 2008; Rajendran *et al*., 2012; Andrade *et al*., 2013; Shwab *et al*., 2014; Rêgo *et al*., 2017; Bernstein *et al*., 2018; Valenzuela-Moreno *et al*., 2022*a*). On the other hand, we discovered the ToxoDB #38 genotype in Campeche, which has only been found in Colombia, where it has been isolated from dogs, cats and chickens (Dubey *et al*., 2006, 2007; Rajendran *et al*., 2012).

Considering all typed markers, there is a predominance of type I and III alleles (with the exception of ToxoDB #116, which has a type II allele at *c22-8*). Our results are in agreement with most of the isolates that have been obtained in the Mexican Neotropical region and with what has been reported from Central and South American countries with a predominance of strains related to genotypes I and III but few or no type II variants (Rajendran *et al*., 2012; Valenzuela-Moreno *et al*., 2020, 2022*a*; Rico-Torres *et al*., 2023)

For the virulence markers, we identified three *ROP18*/*ROP5* virulence profiles in 7 samples (3 tissue samples and 4 *T. gondii* isolates), 2 of which had predicted high virulence in a murine model (4/1 and 1/3) and one with unknown virulence due to the new *ROP5* combination (3/1&3). The virulence profile of the strains from the heart of chicken #1, TgCkMxCam1a and TgCkMxCam1b coincides with the profile described for the other ToxoDB #38 isolates from Colombia (TgCtCo4, 10, 11 and TgDgCo17), a genotype that remains unaltered for the 5 PCR-RFLP virulence markers, despite the locations being over 2400 km away from each other and more than 17 years having elapsed since the profile was first described (Dubey *et al*., 2006, 2007). It would be interesting to perform microsatellite analysis to define how related these strains are.

The profile of the ToxoDB #116 strains described here is different from that previously reported in México because the latter has a *ROP5* type 3 allele and the strains described here have the recombinant 1&3 allele. This *ROP5* result is exceptional, not previously reported, and it could be due to a mixed infection of two strains carrying alleles 1 and 3, but the same result was obtained in the 2 isolates and the clinical samples from the dog #8. In addition, the RFLP results of the other 14 genotyping markers did not show evidence of mixed infection. The *ROP5* locus is an unusual segment of the *T. gondii* genome formed by tandem paralogous genes: *ROP5a*, *ROP5b* and *ROP5c*. These paralogous genes have different repeats and arrays between the 3 archetypical strains (Reese *et al*., 2011). Therefore, it is possible that during recombination events in the intestine of a cat, intragenic recombination occurred at the *ROP5* locus, and the dog #8 ingested a strain with the recombinant *ROP5* 1&3 locus. The ToxoDB #116 genotype carries a *CS3* and *ROP18* type III allele (avirulent in mice); however, the role of the recombinant 1&3 *ROP5* allele in the virulence of *T. gondii* is still unknown (Pena *et al*., 2008). On the other hand, ToxoDB #38 isolates have a type I allele at the *CS3* marker that has been associated with high virulence in the murine model and correlates with the prediction of *ROP18*/*ROP5*. Finally, we found the combination 4/1 *ROP18*/*ROP5* again in México. This fact is highly significant because this pattern can cause up to 100% mortality in the murine model, and it has only been reported a couple of times previously, suggesting that some Mexican strains could pose a high risk to the human and nonhuman animal populations (Shwab *et al*., 2016; Rico-Torres *et al*., 2023).

In conclusion, ToxoDB #38 and #116 genotypes were found in free-range chickens and stray dogs, respectively. The latter variant presents an atypical virulence genotype not previously reported and could be endemic to this region of México. Of the samples that could be genotyped for virulence markers, two profiles would be of high virulence, and one of low virulence. It will be crucial to determine their phenotypes to corroborate the virulence profiles of the *T. gondii* isolates.

## Data Availability

This section is only required if data is available elsewhere. Data Availability Statements are brief statements telling readers how they can access the data and other materials that would be necessary to replicate the findings of an article, in the interests of research transparency.
